# What the pediatric nurse needs to know about the Impella cardiac assist device

**DOI:** 10.1177/02676591241237761

**Published:** 2024-04-23

**Authors:** Giorgia Borrelli, Ilaria Nittolo, Chiara Bochicchio, Laura Trainelli, Valerio Confalone, Tiziana Satta, Federica Cancani, Richard Kirk, Antonio Amodeo, Matteo Di Nardo

**Affiliations:** 1 Pediatric Intensive Care Unit, Bambino Gesù Children’s Hospital, IRCCS, Rome, Italy; 2 Academic Department of Pediatrics (DPUO), Immune and Infectious Diseases Division, Research Unit of Primary Immunodeficiencies, Bambino Gesù Children’s Hospital, IRCCS, Rome, Italy; 3 Heart Failure, Transplantation and Cardio-Respiratory Mechanical Assistance Unit, Bambino Gesù Children’s Hospital, IRCCS, Rome, Italy

**Keywords:** pediatric heart failure, pediatric ventricular assist device, temporary mechanical circulatory support

## Abstract

**Background:** Cardiogenic shock in children still carries a high mortality risk despite advances in medical therapy. The use of temporary mechanical circulatory supports is an accepted strategy to bridge patients with acute heart failure to recovery, decision, transplantation or destination therapy. These devices are however limited in children and extracorporeal membrane oxygenation (ECMO) remains the most commonly used device. Veno-arterial ECMO may provide adequate oxygen delivery, but it does not significantly unload the left ventricle, and this may prevent recovery. To improve the likelihood of left ventricular recovery and minimize the invasiveness of mechanical support, the Impella axial pump has been increasingly used in children with acute heart failure in the last decade. **Purpose:** There are still limited data describing the Impella indications, management and outcomes in children, therefore, we aimed to provide a comprehensive narrative review useful for the pediatric nurses to be adequately trained and acquire specific competencies in Impella management.

## Introduction

Indications for mechanical circulatory support (MCS) in children include heart failure due to congenital heart disease, cardiomyopathy, myocarditis and cardiac allograft failure. Unfortunately, therapeutic options to manage these clinical situations are mainly limited to extracorporeal membrane oxygenation (ECMO).^1,2^ Recent data suggest that the temporary MCS Impella (Abiomed, Danvers, MA, USA), used in adults for the management of cardiogenic shock, can be safely used in children with acute heart failure.^1,3^ However, these patients must have specific anatomic condition including an adequate length of the left ventricle (LV) and of the ascending aorta and, an adequate peripheral vascular access diameter to grant optimal functioning of the device.^4^

Based on these considerations and on the increasing use of the Impella device in children, the pediatric nurse should be adequately trained and acquire specific competencies in Impella management.

## The impella system

**Figure 1.**
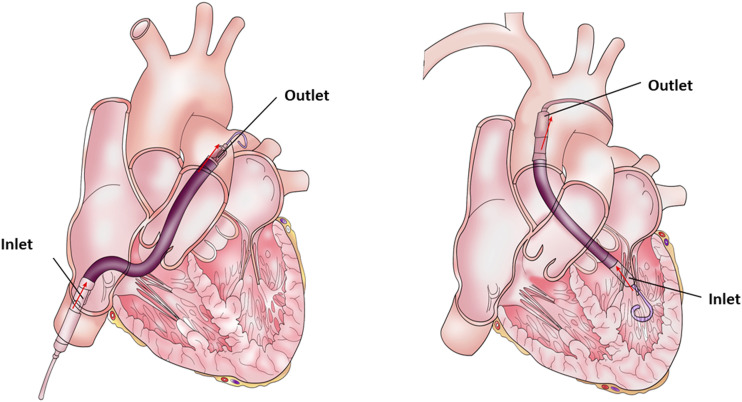
Common sites of Impella catheter position.

The Impella catheter is a micro-axial pump that delivers blood from the LV to the ascending aorta or from the right ventricle (RV) to the pulmonary artery (Impella RP) (Figure 1). The Impella catheter can be inserted percutaneously or via surgical cut-down into the LV to provide systemic perfusion and/or LV unloading when associated with ECMO. In this review we will focus mainly on the Impella for the LV support, however, many of the principles discussed here can be used when using the Impella RP for management RV failure.^5–7^

Currently, there are four Impella catheters (the Impella 2.5, CP, 5.5 and LD) to provide LV support (Table 1).^8^ All catheters have a similar structural design and incorporate a 30° bend (except the Impella LD). The tip of the Impella catheter is generally constituted of a flexible 6 French (F) pigtail to avoid LV injury during placement (the pigtail is absent in the models LD and 5.5). The pigtail ends at the “teardrop”, a radiopaque/echogenic structure located before the inlet area of the catheter. This part of the catheter drains blood from the LV. The outlet part of the catheter contains the motor housing and releases the blood into the ascending aorta.

**Table 1.** General characteristics of Impella catheters.

	Impella 2.5	Impella CP	Impella CP with SmartAssist	Impella 5.5 with SmartAssist	Impella LD	Impella RP
Flow rate (L/min)	2	3.5	3.5	5	4	4
Motor diameter	12F	14F	14F	19F	21F	22F
Insertion method	Percutaneous	Percutaneous	Percutaneous	Surgical	Surgical	Percutaneous
Cannula depth	3+/−0.5 cm	3+/−0.5 cm	3+/−0.5 cm	4.5+/−0.5 cm	4.5+/−0.5 cm	2+/−0.5 cm

The Impella 2.5 has two lumens in the outlet part of the catheter. One lumen is dedicated for the aortic pressure monitoring, while the other lumen continuously purges the motor to prevent the formation of thrombus. The catheters with “SmartAssist” function (CP and 5.5), instead, have a pressor sensor that generates an electrical signal (“placement signal”) proportional to the difference between the pressure outside and inside the Impella. When the Impella is placed in the correct position across the aortic valve, the top (outer surface) of the pressor sensor is exposed to the pressure in the ascending aorta and the bottom (inner surface) of the sensor is exposed to the pressure in the left ventricle. Therefore, the placement signal provides a pressure that is a surrogate of the pressure difference between the ascending aorta and the left ventricle.

The “motor current waveform” is a measure of the energy intake of the Impella catheter. This energy varies with the motor speed and the pressure difference between the inlet (LV) and the outlet (aorta) pressures of the catheter. When the Impella is positioned correctly, the motor current waveform is pulsatile. Both the placement signal and the motor current waveforms are used to check the Impella position during placement and for monitoring the catheter position during extracorporeal support.

The Impella blood flow depends on the axial pump support level (“*p*” level) which is set by the physician and by the pressure gradient between the ascending aorta and the left ventricle. The *p* levels vary from 0 to 9. As a general rule, the physician selects the lowest P-level (P-2 or higher) to provide adequate systemic perfusion (Table 1 Supplement).

The Impella catheter connects to an automatic controller that provides an interface for monitoring the position and the function of the catheter and, the purge solution into the Impella catheter. At the top of the screen is displayed the alarm window. The color of the alarm corresponds with the alarm priority (red: critical; yellow: serious; white: advisory; gray: previously resolved alarm). At the bottom of the screen is displayed the Impella flow, the purge system velocity and the system power.

The automatic controller comprehends also a backup power when the device is used away from the alternating current (AC) power.

The purge cassette delivers a purge solution (typically 5% dextrose solution) to the axial pump to prevent blood from entering into the motor and clotting. The velocity of the purge solution is automatically controlled by the Impella software to keep the purge pressure around 600 mmHg. The amount of purge solution administered per hour can only be quantified retrospectively.

## Pediatric anthropomorphic and anatomic factors to determine impella candidacy

In adults, the Impella 2.5 and CP are generally inserted percutaneously into the femoral artery under fluoroscopic and echocardiographic guidance in the catheterization laboratory; the Impella 5.5, is positioned by surgical cutdown using a prosthetic graft sewn onto the subclavian artery.

In children, the most important factors to determine patient candidacy for Impella placement are: (a) LV length, (b) ascending aorta length (c) the diameter of the vascular access.^4^ Notably, the minimum arterial diameter necessary for the insertion of Impella 2.5 is 4.5 mm^4^, thus, small femoral vessels and the presence of pre-existing vascular occlusions from previous catheterizations can limit its insertion. Based on these considerations, alternative access sites have been reported in children (axillary, carotid artery, etc.) to facilitate the Impella insertion.^9–11^ Furthermore, several authors have also proposed the use of a chimney graft (6 mm diameter) anastomosed to the femoral/axillary/carotid artery to allow Impella placement in children.^9,12,13^ The smallest patient supported with an Impella 2.5 was a 3.5 years old child (12 kg body weight, 0.57 m^2^ body surface area) with a chimney graft on the axillary artery.^14^

**Figure 2.**
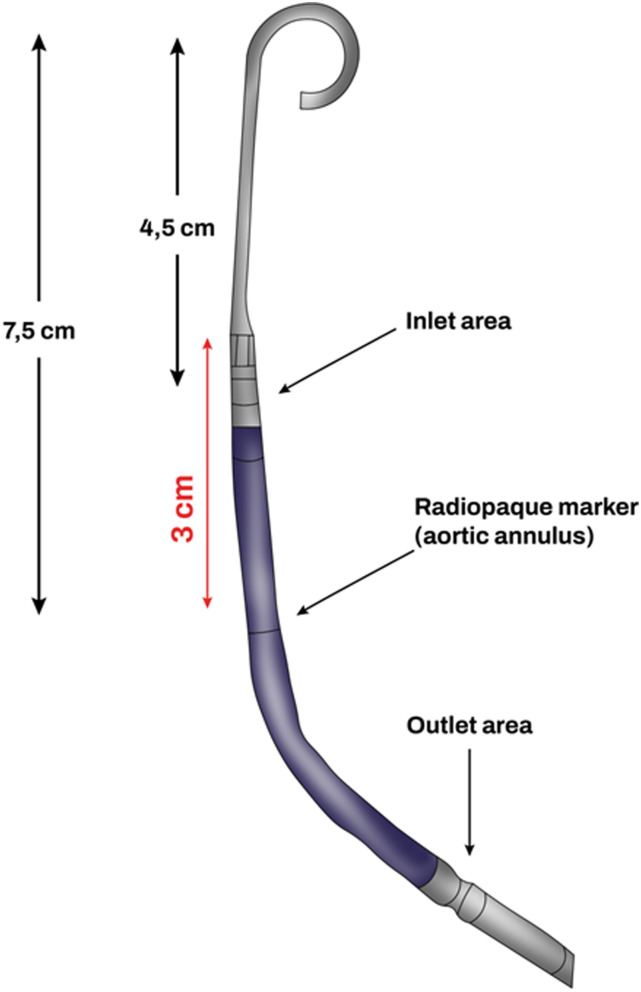
Impella 2.5 catheter: size and characteristics.

Another important point to consider when evaluating Impella candidacy is the distance from the pigtail to the aortic annulus marker. This distance is 7.5 cm in the Impella 2.5, thus, the minimum LV dimension (apical length) to allow the device to work in an unconstrained fashion is 7.5 cm (Figure 2). Bending the soft portion of the same pigtail in the ventricular apex, can shorten the functional length down to 5.5 cm, and can expand the candidacy options.^4^

Morray et al.,^4^ using echocardiographic and magnetic resonance imaging sequences of the LV of children with and without cardiac disease, showed that a LV length of 7.5 cm corresponded to a typical patient with a height of 122 cm, a weight of 23 kg and a body surface area of 0.89 m^2^.

## How to check the impella catheter position

**Figure 3.**
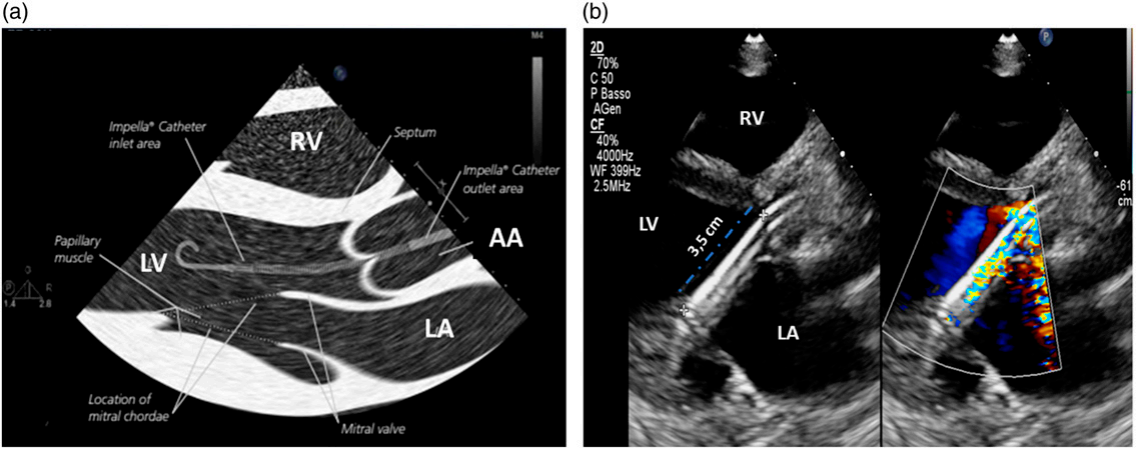
(a) Work-art representation of the ultrasound anatomy a transthoracic parasternal long-axis view of the left ventricle (courtesy of Abiomed, Danvers, MA, USA); (b) Transthoracic parasternal long-axis view of the left ventricle: evaluation of the Impella position (3.5 cm distance from the tear drop to the aortic annulus). RV: right ventricle, LV: left ventricle; LA: left atrium; AA: ascending Aorta.

The Impella catheter can be placed in different anatomical sites: femoral artery, innominate artery, axillary artery and carotid artery,^1,3^ however, regardless of the access site, the Impella catheter must be properly positioned (Figure 3(a)) to provide optimal hemodynamic support and minimize the risk of complications including hemolysis, mitral valve apparatus damage and arrhythmias.

The Impella position should be checked daily or when the monitor alarms or the device malfunctions. The correct position of the inlet part of the Impella is in the LV, while the outlet part is in the ascending aorta. Notably, the Impella catheter must be oriented towards the anteroseptal portion of the LV to avoid mechanical injuries to the mitral valve apparatus.

**Figure 4.**
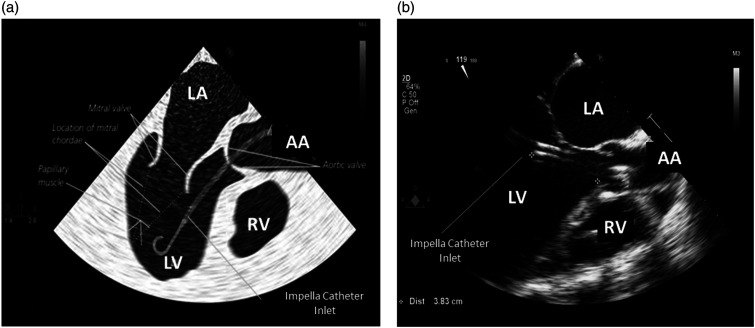
(a) Work-art representation of the ultrasound anatomy of a transesophageal mid-esophageal long-axis view (120°) (courtesy of Abiomed, Danvers, MA, USA); (b) Transesophageal mid-esophageal long-axis view (120°) of an Impella CP positioned at 3.83 cm from the aortic annulus. RV: right ventricle, LV: left ventricle; LA: left atrium; AA: ascending Aorta.

Cardiac ultrasound is the best technique to assess the position of the Impella catheter and, if necessary, reposition it, because other techniques (e.g., chest X-ray) do not allow for accurate Impella visualization within the cardiac structures.^15^ The transthoracic parasternal long-axis view is the most common acoustic window used to check the Impella position (Figure 3(b)). Transesophageal echocardiography (120° mid-esophageal long-axis view) is generally used for catheter placement in the catheterization laboratory (Figure 4(a) and (b)).^16^

**Figure 5.**
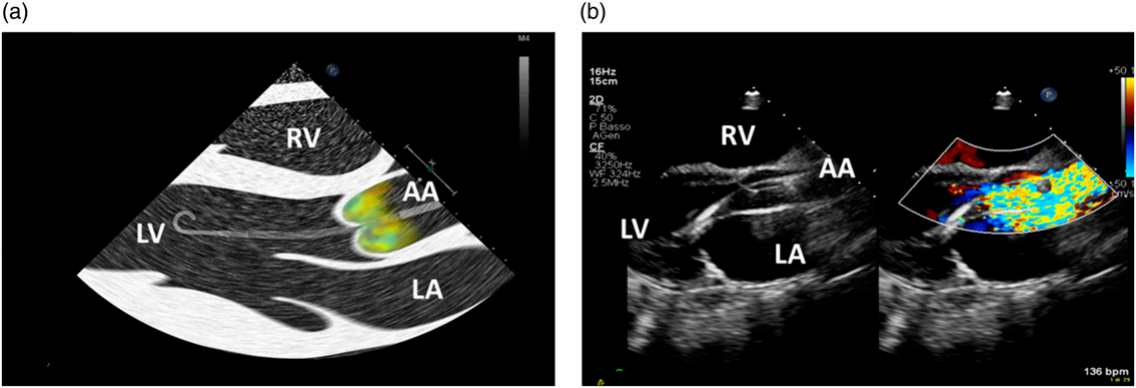
(a) Work-art representation of the ultrasound anatomy a transthoracic parasternal long-axis view of the left ventricle showing turbulence in the ascending aorta (Impella outlet); (b) Transthoracic parasternal long-axis view of the left ventricle showing with color Doppler the correct position of the outlet part of the Impella CP catheter above the aortic annulus. RV: right ventricle, LV: left ventricle; LA: left atrium; AA: ascending Aorta.

With the transthoracic parasternal long-axis view, the Impella catheter appears as two echogenic parallel lines crossing the aortic valve towards the LV (Figure 3(b)). The optimal catheter depth, defined as the distance from the aortic annulus to the teardrop, is 3 ± 0.5 cm for the Impella 2.5 and CP, while is 4.5 ± 0.5 cm for the Impella 5.5. When the catheter is not correctly positioned, repositioning should be performed under echocardiographic guidance with two operators, one performing the cardiac ultrasound and the other manipulating the catheter. During this procedure the power of the device should be set to the lowest level of support (P2) to avoid injury of the mitral valve apparatus. Color Doppler may further confirm catheter position. If the Impella catheter is correctly positioned, a dense mosaic pattern of turbulence will appear above the aortic valve near the outlet area of the catheter (Figure 5). If, the dense mosaic pattern of turbulence is beneath the aortic valve, this indicates that the outlet area is wrongly positioned (too far into the LV or entangled in papillary muscle). Cardiac ultrasound can be also used to identify potential contraindications to the Impella catheter placement such as the presence of: a mechanical aortic valve, aortic dissection, moderate to severe aortic valve regurgitation or stenosis, LV thrombus, LV infarction or rupture, and, obstructive or hypertrophic cardiomyopathy.^2^

## How to assess and change access site dressing

The nursing care of the access site is an essential part of the daily Impella catheter management and starts immediately after its insertion in the operating theatre (Table 2 Supplement).

**Figure 6.**
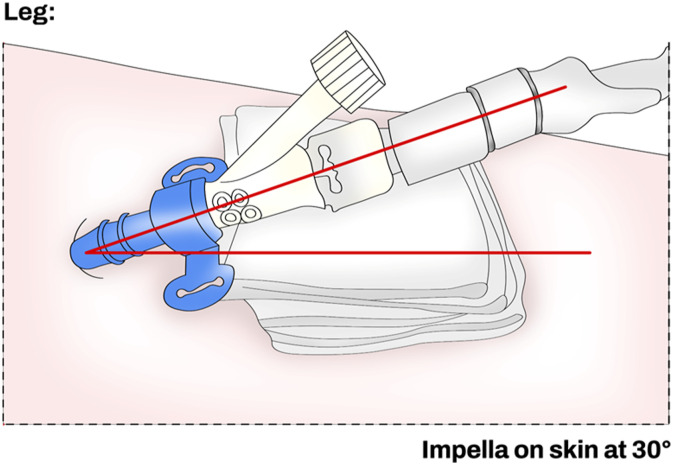
Correct position of the Impella sheath when inserted percutaneously.

The Impella access site should be always clearly visible and the angle of entry of the device should be stabilized using an external support (e.g., 4*4 folded gauze) during the transportation to the pediatric intensive care unit (PICU) (Figure 6). When in PICU, the nurse in charge of the patient should be always aware of the type of the Impella catheter implanted and if the peel-away sheath has been removed before leaving the operating theatre. If the peel-away sheath is left in situ, the patient may have a higher risk of bleeding, damage to the arteriotomy, limb ischemia, or clots within the peel-away sheath.

If bleeding or oozing occurs, the nurse should remove the dressing and clean the access site as is standard practice for any arterial line, and the new dressing should be applied as per hospital protocol with an aseptic technique. If bleeding or oozing at the access site persists, the nurse should notify it to the physician and maintain the entry angle of the Impella with gentle pressure (Table 3 Supplement). Common interventions to prevent the access site bleeding include: the use of a knee immobilizer, the maintenance of the head of the bed at less than a 30-degree angle, minimizing unnecessary movements, patient comfort and, ensure that the Impella catheter is not pulled away during transfers or when the patient is cleaned (Table 4 Supplement). At the end of a dressing change, the nurse should always check the angle of entry of the catheter, ensure the centimeter markers on the catheter shaft are visible and the Tuohy-Borst valve accessible. To maintain catheter visibility, use two transparent dressings and cut a triangle into each of them to fit over the insertion site.

## Anticoagulation management for impella

The optimal functioning of the Impella catheter requires both intra-pump and systemic anticoagulation. Intra-pump anticoagulation is required to prevent clots in the motor and is performed with the infusion of a heparinized dextrose purge solution, while systemic anticoagulation is required to prevent clots around the Impella catheter and is generally performed with heparin.^17^

**Figure 7.**
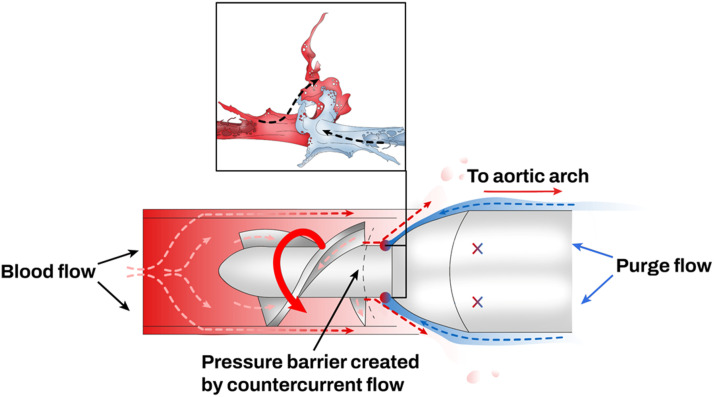
Purge solution (blue) counter-current to the blood flow (red).

The purge solution runs counter-current to the blood flow through the pump, prevents blood from reaching the motor assembly,^17^ and ends in the systemic circulation via the outlet area (Figure 7).^8^ The purge solution is generally constituted of dextrose 5% and heparin. Heparin increases the pH of the dextrose purge fluid and stabilizes blood proteins preventing their deposition and aggregation in the motor. Without heparin in the purge solution, blood proteins may deposit into the motor leading to a high motor current, friction and hemolysis.

Reed et al.^18^ conducted a survey in USA centers using the Impella catheter and showed that different heparin purge concentrations (ranging from 0 units (U)/mL to 50 U/mL) are used and that 41% of these centers do not adjust the dose of systemic heparin in relation to the dose of heparin in the purge solution. Additionally, dextrose concentration also varied among centers (between 5% and 20%). These heterogeneous practices may complicate the management of systemic anticoagulation and increase the risk of bleeding. The manufacturer currently recommends a heparin concentration of 50 IU/mL in a 5% dextrose solution, nevertheless, various dextrose solutions have been used (ranging from 5% to 40% dextrose concentration) in clinical practice. In general, lower dextrose concentrations (5%) are less viscous and result in a fast flow rate through the purge system and more systemic heparin exposure; conversely, higher dextrose concentrations are more viscous and result in a slower purge flow rate and less systemic heparin exposure for the patient.^17^

Despite being rare in children,^19^ the use of heparin may be associated with the heparin-induced thrombocytopenia (HIT). In these cases, systemic anticoagulation can be achieved with the use of direct thrombin inhibitors (e.g., bivalirudin and argatroban), while intra-pump anticoagulation can be achieved using a bicarbonate-based purge solution.^20,21^ The bicarbonate works by increasing the pH of the dextrose solution in the same way as heparin and prevents clot formation in the motor. The bicarbonate-based purge solution (sodium bicarbonate 8.4% 25 mEq in 1 L dextrose 5%) has been approved by the Food and Drug Administration in US as an alternative to heparin in patients with HIT.^20^ Bashline et al.^21^ showed in their single center retrospective study including 34 patients supported with Impella that the use of systemic bivalirudin and bicarbonate-dextrose purge solution was associated with a rate of adverse events similar to the one previously reported with heparin. Recently, Al-Ayoubi et al.^20^ also showed the successful use of the bicarbonate–dextrose purge solution and bivalirudin for systemic anticoagulation in two patients with cardiogenic shock who developed HIT. They found no significant change in the Impella purge pressure or flow overtime in both patients. Currently, there are no data on the use of the bicarbonate-based purge solution in children.

Regardless of the type of anticoagulation, the manufacturer recommends starting anticoagulation via the purge fluid in the catheterization laboratory or as soon as the patient is in PICU. The suggested anticoagulation target are: an activated clotting time between 160 and 180 s, a heparin anti-factor Xa between 0.3 and 0.5 when using heparin or an activated partial thromboplastin time of 60–90 s when using bivalirudin.^15^

In general, anticoagulation goals are not met with the purge solution alone, therefore, systemic anticoagulation needs to be added. However, if the patient is excessively anticoagulated only with the purge solution, the manufacturer suggests switching to half concentration of heparin (12.5 U/mL) in 5% dextrose concentration to minimize the heparin exposure.

## What to look at bedside

The nursing management of a pediatric patient supported with Impella is challenging and includes not only the monitoring of the patient’s vital parameters (e.g., heart rate, blood pressure, diuresis, pulse oximetry, temperature, refill time, etc.), but also interpreting the alarms and the Impella waveforms on the automated Impella controller.

**Figure 8.**
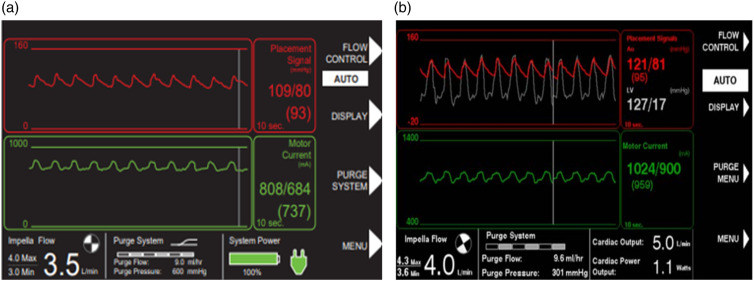
Automated Impella Controller showing: (a) correct Impella position; (b) correct Impella position (with SmartAssist).

**Figure 9.**
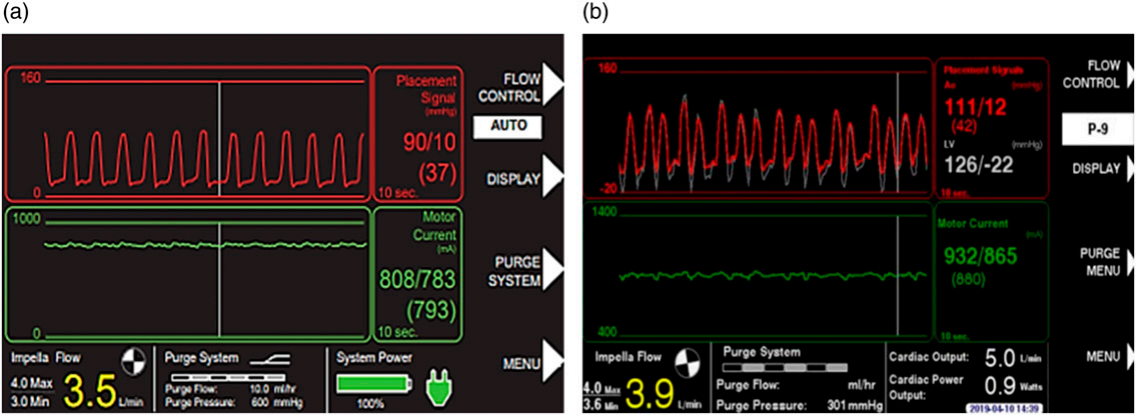
(a) Impella position wrong: catheter in the left ventricle; (b) Impella position wrong: catheter in the left ventricle (with SmartAssist).

**Figure 10.**
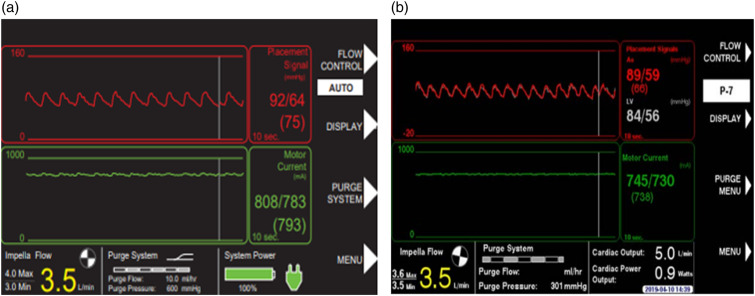
(a) Impella position wrong: catheter in the ascending aorta; (b) Impella position wrong: catheter in the ascending aorta (with SmartAssist) (courtesy of Abiomed, Danvers, MA, USA).

When the Impella is correctly positioned, the placement signal and the motor current are pulsatile (Figure 8(a) and (b)), but when the Impella catheter is malpositioned or dysfunctional the controller will alarm (Figures 9 and 10). There are two types of positional alarm: the “Impella position wrong” and the “Impella position unknown”. The first one occurs when both the placement signal and motor current have low pulsatility. In this case, the Impella catheter (inlet and outlet) could be either in the LV (Figure 9(a) and (b)) or in the ascending aorta (Figure 10(a) and (b)). In presence of low heart pulsatility, the Impella controller cannot identify the correct catheter position, thus, it will display either the alarm “Impella position unknown” or the “Impella position wrong”. Cardiac ultrasound may help to accurately interpret these two alarms.

**Figure 11.**
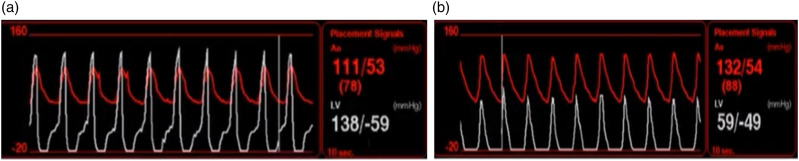
Suction Alarm. (a) Impella low flow associated to a negative diastolic pressure that recovers by the end of diastole, combined with normal systolic pressure (left ventricle) suggests hypovolemia. (b) Impella low flow associated to a negative diastolic pressure that does not recover at the end of diastole, combined with a low systolic pressure (left ventricle), suggests impaired device positioning.

Additionally, the nurse should be able to interpret the “suction alarm” (hypovolemia vs malposition) (Table 5 Supplement). If the LV shows a negative diastolic pressure with normal systolic pressure, the patient may be hypovolemic (Figure 11(a)). If the LV shows a low systolic pressure and a negative diastolic pressure, incorrect position is more likely the reason of alarming (Figure 11(b)).

## Complications associated with use of impella

The most common complications associated with the use of Impella are: hemolysis, bleeding at the insertion site, arrhythmias, thrombocytopenia, aortic valve or mitral apparatus injury, vascular injury, limb ischemia, device malfunction, site infections and acute brain injury (embolic stroke).^22^

Hemolysis has a pediatric occurrence rate of 8% (similar to that reported in adults supported with Impella for cardiogenic shock) and may be due to both device and patient-related factors.^1,23^ In general, hemolysis can be due either to a partial obstruction of the inlet/outlet part of the catheter (clots, interference with the mitral valve apparatus, etc.), to suboptimal cardiac device position or, to the use of high P-levels (high erythrocyte shear stress). Reduction of the purge solution flow or obstruction may also increase the risk of intra-motor thrombosis and hemolysis. Patient anatomical characteristics can also contribute to hemolysis. Nakamura et al.^24^ showed that a narrow angle (<126.5°) between the aortic and mitral annulus on transthoracic echocardiography is an independent risk factor for refractory hemolysis in adults. This narrow angle may potentially orient the pump towards the lateral or posterior wall of the LV resulting in increased suction and obstruction.

Hemolysis should be daily monitored during Impella support because it can increase the risk of acute kidney injury and multiorgan failure^25^ (Table 6 Supplement). An increase of plasma-free hemoglobin (>40 mg/dl) may be suggestive of hemolysis as well as high lactate dehydrogenase levels, decreased hemoglobin levels, micro/macro hematuria, and in some cases, acute renal failure.^15^ The pediatric nurse should be aware of all these complications and immediately notify to the physician if they occur.

Bleeding and thrombotic complications are other causes of morbidity and mortality associated with the use of Impella. Thrombosis may be induced by the critical illness itself and by the non-biologic material of the pump.^23^ In contrast, bleeding may be due to the development of an acquired von Willebrand syndrome due to high shear stress and reduced blood pulsatility.

This phenomenon is also facilitated by an improper Impella malrotation inside the LV. Baldetti et al.^26^ observed that the malrotation of the Impella catheter inside the LV was associated with a significant increase of bleeding events. Notably, both the presence of liver failure (low level of coagulant factors) and the use of high level of systemic anticoagulation may further aggravate the risk of bleeding. Access site bleeding may be also due to the insertion of a large sheath size, the accidental patient mobilization with the loss of the angle of entry of the catheter or to the use of excessive anticoagulation. Using an arterial coil after Impella removal has been described as a feasible strategy to reduce the risk of bleeding.^27^

Ventricular arrhythmias are complications that can occur during Impella positioning or after its placement if the catheter is not in the correct position. Thus, frequent assessment with cardiac ultrasound of the Impella position may reduce the risk of arrhythmias and cardiac injuries including pericardial effusion, aortic dissection, mitral valve apparatus damage, etc.^28^

When arrhythmias are not related to the catheter position both cardiopulmonary resuscitation (CRP) and defibrillation can be done without stopping the device; however, if CRP is needed, the level of Impella assistance should be reduced to P2 to avoid cardiac injuries (Table 7-8 Supplement).^29^

Limb ischemia is another potential complication associated with the use of the Impella catheter (Table 9 Supplement). The pediatric nurse should frequently monitor the development of limb ischemia by checking the distal arteries pulsatility, the different temperature between the two legs, the skin color and the perfusion of to the leg with the near infrared spectroscopy (NIRS). To reduce the risk of limb ischemia, a dedicated perfusion catheter or a 14F sheath modified with two perfusion holes in the dorsal sheath surface^30,31^ may be used; in alternative, in case of small femoral arteries, a chimney graft enveloping the sheath may be used.^11^ Nevertheless, when limb ischemia is refractory to any treatment, device explant should be immediately considered.

The occurrence of stroke is rare in children supported with Impella,^1,3^ however, embolization of clots around the pump may happen and should be properly monitored.^22^

## Transport with impella

In-hospital, helicopter and fixed wing transportation can be safely done in patients supported with Impella.^29,32^ However, the pediatric nurse must be aware of some important aspects (Table 10 Supplement) and when the Impella is combined with ECMO (ECPELLA), the ECMO transport guidelines from the Extracorporeal Life Support Organization should be also followed.^33^

Before attempting a transport of a pediatric patient supported with Impella, the nurse of the transport team should be prepared to manage these three important points: (a) the battery charge of the automated Impella controller, (b) where and how to secure the automated Impella controller during transport and c), the potential presence of air in the purge solution bag.^29^

The automated Impella controller works with an AC of 100–230 V, however, it can also run on battery power alone (internal battery 14.4 V) for a maximum of 60 min. Thus, for long transportation, it is mandatory to use a built-in direct current to AC power inverter.

Inside the transport vehicle, the automated Impella controller should be fixed to a flat and secure surface and, should be clearly visible to all team members to fix alarms and make any necessary changes. Its cooling vents should not be blocked to avoid malfunctioning and pump failure. When possible, the Impella catheter should be properly secured to the patient and the connector cable from the controller to the Impella catheter should not be stressed since tension could move the catheter out of the correct position and compromise patient circulatory support. Importantly, when transferring a patient with the Impella device in place, the head of the patient’s bed should not be raised higher than 30° degrees because this action may increase the risk of bleeding at the access site especially if a peel-away sheath is left on site.

The purge solution bag should be de-aired before starting a transport to reduce the need for removing air from the Impella system during transport. Notably, the purge pressure should be always monitored during helicopter transport since it can change with altitude.

## ECPELLA

The Impella catheter can be also used combined with VA ECMO (ECPELLA or ECMELLA) to provide LV unloading. There is little experience of ECPELLA in children with acute heart failure, however, the initial data have been promising.^11,12^ Different approaches have been proposed in children to provide LV unloading during ECMO, with percutaneous atrioseptostomy the most studied.^34^ Nevertheless, a recent simulation study comparing Impella versus atrioseptostomy for LV unloading showed the superiority of Impella because it provides both cardiac unloading and hemodynamic support.^35^

When ECMO and Impella are used together, different arterial access sites are generally used. However, an innovative method to introduce both the Impella and arterial ECMO cannula through the same arterial access site has been successfully described.^36^

Management of ECPELLA (the concomitant use of both VA ECMO and Impella) is challenging. While VA ECMO is used to provide end-organ perfusion and oxygen delivery, Impella is introduced to reduce LV filling pressure. The Impella is initiated at the minimum P-level to provide LV unloading, then, when end-organ dysfunction improves a reduction of the ECMO flow is attempted while increasing the Impella support.^37,38^ A careful flow balance between the two devices should be maintained to provide a rapid cardiac function recovery and to avoid the development of hemolysis and ventricular arrhythmias. When ECPELLA is used, ECMO is generally weaned before Impella since it is associated with more complications,^15^ finally, when hemodynamic stability is achieved after ECMO withdrawal (adequate diuresis, mean arterial pressure, decrease lactate levels, mixed venous oxygen saturation >70%), Impella weaning is started.^37^ Right ventricular dysfunction represents the major limit for moving from biventricular support to univentricular support.

## BIPELLA

Patients with severe biventricular dysfunction after heart transplantation are generally treated with inotropic support and immunosuppressive therapies, however, when medical therapy fails, VA ECMO can be used to improve systemic perfusion while reducing myocardial oxygen consumption. Temporary MCS with Impella has been successfully used as an alternative to ECMO to manage biventricular dysfunction due to their more favorable adverse events profile and the need of lower levels of anticoagulation. The Impella catheter can support both the LV and RV. The Impella RP has been used in adults to support the RV and its use has increased significantly after the results of the Recovery Right study^39^ which showed efficacy and safety of this device. Recently the FDA approved the use of Impella RP in children with a BSA >1.5 m^2^ with RV failure after VAD implantation or rejection after heart transplantation. Currently, few pediatric cases have been supported with bilateral Impella configuration (BiPella) for severe graft dysfunction and all of these were adult sized, nevertheless, the first results were successful and reported few and manageable complications.^5,7^

## Weaning

Once the Impella has been positioned, the pediatric nurse should continuously monitor the patient’s vital signs until the recovery of native heart function. The possibility of weaning from the device should be assessed daily as early as 24–48 h after the initiation of Impella support. In general, weaning should be considered when the patient is stable with a pulsatile arterial waveform, low dose of vasopressors and inotropes. The weaning process is guided by both hemodynamic, cardiac ultrasound and metabolic monitoring (diuresis, lactate levels, skin color, etc.) and consists of a gradual reduction of the level of assistance till the level P2^15^. When the patient is stable at P2 for a reasonable amount of time, the catheter is stopped and removed at beside. Insertion site compression should be done accurately to avoid bleeding.

## Conclusion

The Impella catheter is a temporary MCS that is acquiring popularity in children to support acute heart failure. As with all the new technologies, the bedside nurse should be aware of its indication, functions and moreover, interpret properly all its alarms. Impella-related complications (misposition, LV injury, bleeding, thrombosis, vascular injury, etc.) may be reduced by knowing the device and following specific checklists.

## Supplemental Material

Supplemental Material - What the pediatric nurse needs to know about the Impella cardiac assist device Supplemental Material for What the pediatric nurse needs to know about the Impella cardiac assist device by Giorgia Borrelli, Ilaria Nittolo, Chiara Bochicchio, Laura Trainelli, Valerio Confalone, Tiziana Satta, Federica Cancani, Richard Kirk, Antonio Amodeo and Matteo Di Nardo in Perfusion
