# Supplementary material for: What the pediatric nurse needs to know about the Impella cardiac assist device
Source: Perfusion. 2024 Apr 23;39(1 Suppl):95S–106S. doi: 10.1177/02676591241237761 (PMC11041080; doi:10.1177/02676591241237761)
Supplement: Supplemental Material - What the pediatric nurse needs to know about the Impella cardiac assist device [file sj-pdf-1-prf-10.1177_02676591241237761.pdf]

Checklists

Table 1 Standard assessment for Impella systemic perfusion

• Check and write on the electronic medical chart the Impella P-level
• Check and write on the electronic medical chart the central venous pressure
• Check every 4 hours for lactate levels, the urinary output trend and the skin color
• Notify to the physician if the lactate levels are increasing
• Maintain the lowest Impella P-level to provide adequate systemic perfusion

Table 2 Standard assessment in pediatric intensive care unit of the Impella patient

• Make sure the catheter is clearly visible under the dressing
• Check for adequate placement signal and motor current waveform
• Assess the insertion site
• Mark the Impella for easy assessment of accidental displacement
• Assess distal pulses distal from the insertion site hourly

Table 3 Access site bleeding

• Check for Impella position and sheath type
• Check coagulation status (APTT ratio, INR, platelet count, plasma free hemoglobin levels) and if outside the goal range notify to the physician
• Change dressing frequently when bleeding or oozing
• Control patient comfort
• Minimize leg movement (e.g. knee immobilizer)

Table 4 Patient agitation and discomfort

• Notify to the physician if pain or discomfort is present
• Reposition the patient as needed
• If pain is present, administer analgesics as indicated by the physician

Table 5 Management of "Suction Alarm"

• Decrease P-level to reduce the effect of suction and notify to the physician. *Never drop below P-2 to avoid retrograde flow and be prepared for volume resuscitation*
• Check for hypovolemia and right ventricular dysfunction signs and symptoms (e.g. high central venous pressure, hepatomegaly, low urinary output)
• Prepare for a cardiac ultrasound to evaluate Impella position inside the left ventricle
• When alarm is corrected gradually return to the previous setting

Table 6 Hemolysis Management

• Monitor plasma free hemoglobin and notify to the physician if it is higher than 50 mg/dL
• Control urine output and color (e.g. hematuria)
• Monitor for hypovolemia and notify to the physician
• Monitor Impella position with cardiac ultrasound daily in case of hemolysis
• Reduce P-level in presence of suction

Table 7 Management of ventricular fibrillation/ventricular tachycardia without pulse

• Reduce the Impella level to P2
• Begin cardiopulmonary resuscitation and defibrillate as per pediatric advance life support protocol
• Do not touch the Impella catheter, cables or the automated Impella control during the defibrillation

Table 8 Management of asystole/pulseless electrical activity

• Reduce the Impella level to P2
• Begin cardiopulmonary resuscitation as per pediatric advance life support protocol
• When spontaneous rhythm is regained, check Impella position with cardiac ultrasound and increase the Impella P-level

Table 9 Limb Ischemia management

• Monitor hourly the presence and the quality of the distal pulses in the affected leg
• Evaluate differences in temperature and color between the two legs
• Immediately notify to the physician: absence of pulses, pallor, sensory and/or motor neuropathy

Table 10 Transport of the Impella patient

• Charge the automated Impella control before transport
• Provide a built-in direct current to the AC power inverter if the transport is greater than 60 minutes
• Whenever possible maintain the automated Impella controller connected to the AC power/inverter
• Secure the automated Impella controller to the stretcher during in-hospital transport
• Remove air from the purge fluid bag before any transport
• Ensure that the cooling vents are not blocked

• During vehicle transport, the automated Impella control should be strapped to a flat surface
• Provide during transport an easy access to the display screen of the automated Impella control
• Keep the Impella cables secured and unstressed
• Do not raise the head of the patient’s bed higher than 30 degrees
